# Serum *N*-glycan profiling is a potential biomarker for castration-resistant prostate cancer

**DOI:** 10.1038/s41598-019-53384-y

**Published:** 2019-11-14

**Authors:** Teppei Matsumoto, Shingo Hatakeyama, Tohru Yoneyama, Yuki Tobisawa, Yusuke Ishibashi, Hayato Yamamoto, Takahiro Yoneyama, Yasuhiro Hashimoto, Hiroyuki Ito, Shin-Ichiro Nishimura, Chikara Ohyama

**Affiliations:** 1 Department of Urology, Hirosaki University Graduate School of Medicine, Hirosaki, 036-8562 Japan; 2 Department of Advanced Transplant and Regenerative Medicine, Urology, Hirosaki University Graduate School of Medicine, Hirosaki, 036-8562 Japan; 3 Department of Urology, Aomori Rosai Hospital, Hachinohe, 031-8551 Japan; 4 Graduate School of Life Science, Frontier Research Centre for Advanced Material and Life Science, Hokkaido University, Sapporo, 060-0808 Japan

**Keywords:** Glycobiology, Prostate cancer

## Abstract

We investigated the diagnostic and prognostic potential of serum *N*-glycan profiling for castration-resistant prostate cancer (CRPC). We retrospectively investigated serum *N*-glycan structural analysis by glycoblotting for 287 patients with benign prostatic hyperplasia (BPH), 289 patients with newly diagnosed prostate cancer (PC), 57 patients with PC treated with androgen-deprivation therapy without disease progression (PC-ADT), and 60 patients with CRPC. *N*-Glycan profiling was compared between the non-CRPC (BPH, newly diagnosed PC and PC-ADT) and CRPC patients. We obtained the quantitative score for CRPC (CRPC *N*-glycan score) by discriminant analysis based on the combination of 9 *N*-glycans that were significantly associated with CRPC. The median CRPC *N*-glycan score was found to be significantly greater in CRPC patients than in non-CRPC patients. The CRPC *N*-glycan score could classify CRPC patients with sensitivity, specificity, and area under the curve of 87%, 69%, and 0.88, respectively. The CRPC *N*-glycan score >1.7 points was significantly associated with poor prognosis in patients with CRPC. The glycoprotein analysis showed that not immunoglobulins but α-1-acid glycoprotein (AGP) were a potential candidate for the carrier protein of *N*-glycans. The overexpression of specific *N*-glycans may be associated with their castration-resistant status and be a potential biomarker for CRPC.

## Introduction

Prostate cancer(PC) has been increasing worldwide and major cancer in Japan in recent years^1^. Although prostate-specific antigen (PSA) is a useful biomarker for PC detection^2–4^, the utility of PSA for castration-resistant PC (CRPC) is insufficient^5–9^. Several new prognostic markers, including the clinicopathological status and liquid biopsy (such as circulating tumor cell and cell-free DNA) have been investigated in CRPC^10–13^. Of those, several biomarkers for CRPC using serum glycans have been reported to date^14–16^. More than half of the proteins have glycans and glycans play crucial roles in molecular interactions and signal transduction^17^. As glycosylation for proteins and lipids was tightly controlled by glycosyltransferases, malfunction of the glycan system is related to several diseases and cancers^18–25^. Although cancer-associated glycan alterations represent potential cancer biomarkers, it has not been applied clinically because of the complicated protocols and the analytical methods involved. A new approach that combines chemoselective *N*-glycan enrichment (glycoblotting methods) and quantitative *N*-glycan mass spectrometry improved *N*-glycan analysis^26^. Our previous studies suggested that a quantitative *N*-glycan analysis is a promising approach for biomarker screening in several cancers^14,21,27–30^. Among these studies, a few evaluated the potential diagnostic value of serum *N*-glycomics (tri- and tetra-antennary *N*-glycans) in patients with CRPC^14,21,30^. In the present study, we evaluated the diagnostic and prognostic potential of serum *N*-glycan profiling for CRPC using a combination of serum *N*-glycans.

## Results

Table 1 summarizes the demographics of the study cohort. There were significant differences in age and initial PSA among the groups. The age of patients in the benign prostatic hyperplasia (BPH) group was younger than that in the newly diagnosed PC, PC treated with androgen-deprivation therapy without disease progression (PC-ADT) and CRPC groups. The initial PSA level in the CRPC group was significantly greater than that in the BPH (*P* < 0.001), newly diagnosed PC (*P* < 0.001), and PC-ADT groups (*P* < 0.001).

**Table 1:** Patient Demographics of the Study Cohort.

	Non-CRPC			CRPC
	BPH	Newly diagnosed PC	PC-ADT	
Number of patients	287	289	57	60
Median age (years, IQR)	68 (62–74)	78 (74–83)	78 (74–81)	76 (70–79)
Median initial PSA (ng/mL, IQR)	6.4 (4.9–9.0)	8.6 (5.8–14)	17 (13–38)	101 (21–513)
PSA at *N-*glycan analysis (ng/mL, IQR)			0.04 (0.01–0.24)	50 (6.9–272)

**Figure 1:**
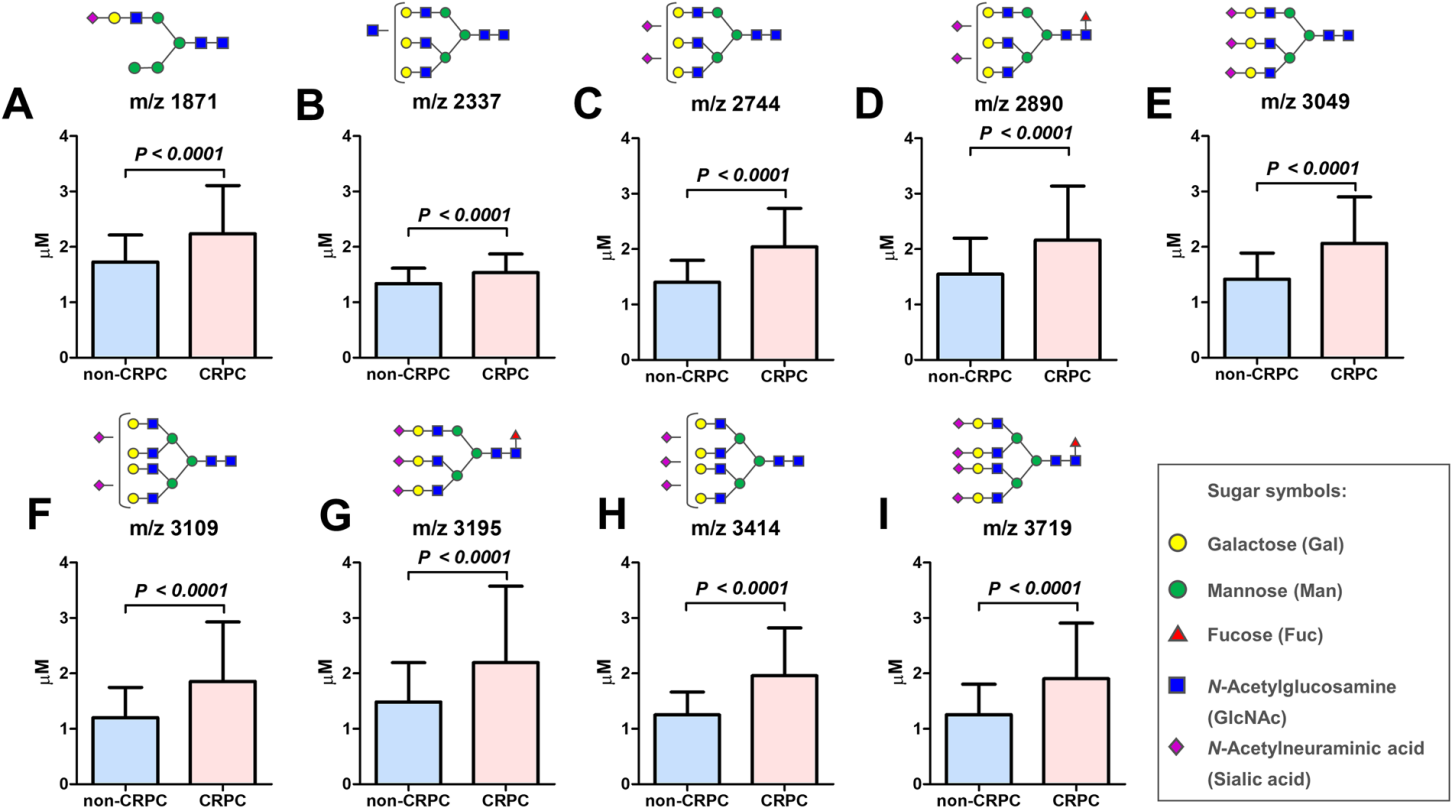
The difference in the serum *N*-glycan levels between the non-CRPC (BPH, newly diagnosed PC, and PC-ADT) and CRPC patients. A significant difference in the serum *N*-glycan levels was noted between the non-CRPC (BPH, newly diagnosed PC, and PC-ADT) and CRPC patients in *m/z 1871* (**A**; *P* < 0.001), *m/z* 2337 (**B**; *P* < 0.001), *m/z 2744* (**C**; *P* < 0.001), *m/z* 2890 (**D**; *P* < 0.001), *m/z* 3049 (**E**; *P* < 0.001), *m/z* 3109 (**F**; *P* < 0.001), *m/z* 3195 (**G**; *P* < 0.001), *m/z* 3414 (**H**; *P* < 0.001), and *m/z* 3719 (**I**; *P* < 0.001).

**Figure 2:**
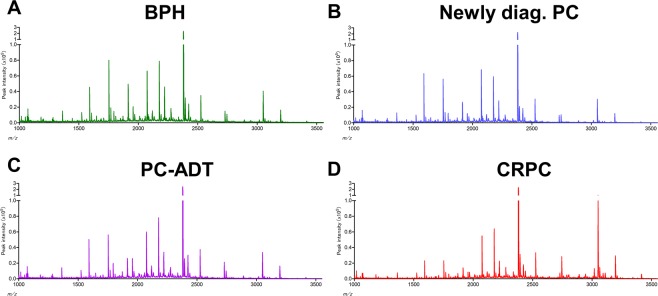
Representative mass spectra. Representative mass spectra were shown in the patients with BPH (**A**), newly diagnosed PC (**B**), PC-ADT (**C**), and CRPC (**D**).

**Figure 3:**
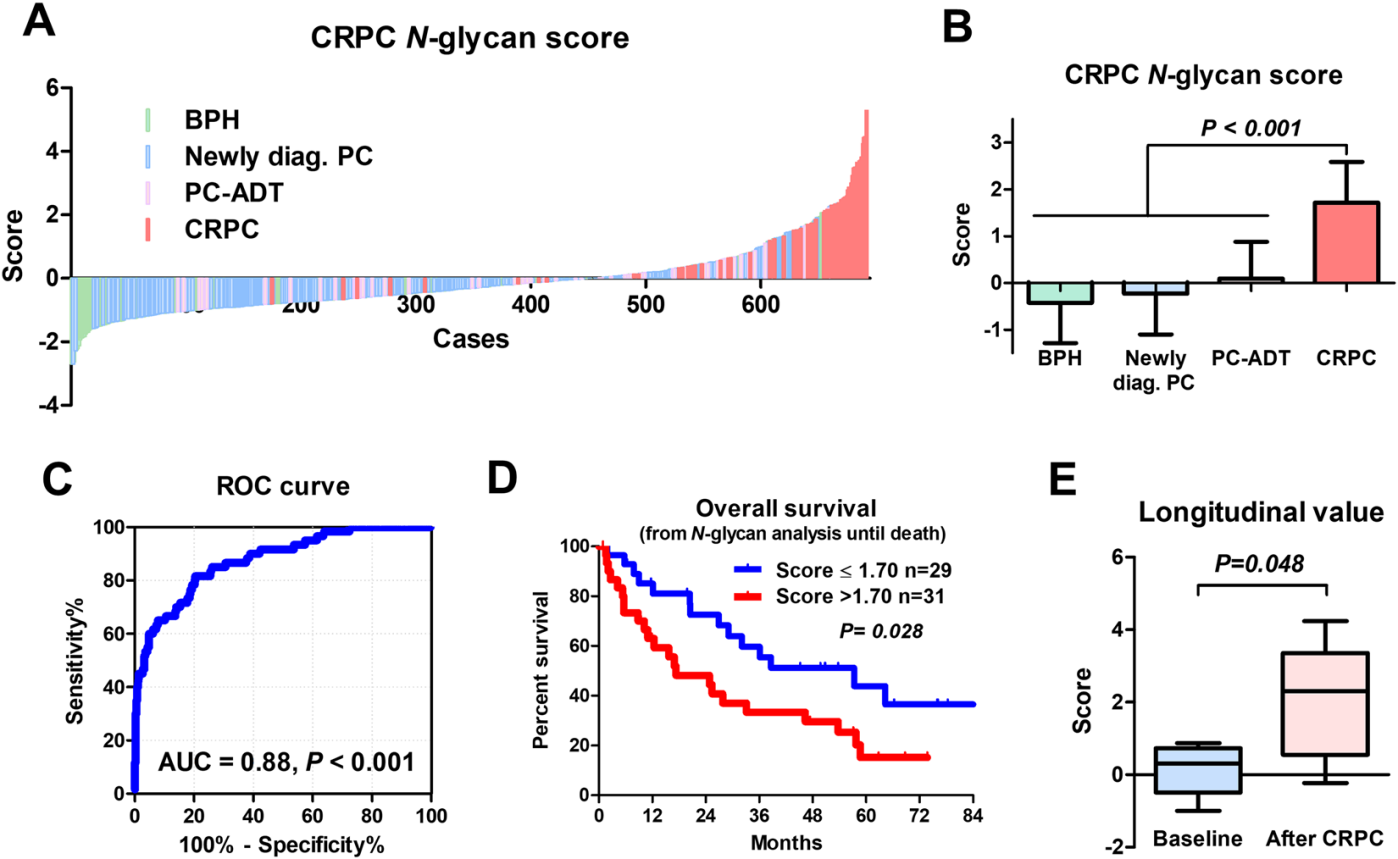
Clinical impact of the CRPC *N*-glycan score on CRPC discrimination. The Waterfall plots show the association between the CRPC *N*-glycan score and the CRPC status (**A**). The CRPC *N*-glycan score was significantly greater in patients with CRPC than in those with BPH, newly diagnosed PC, and PC with ADT (**B**). The predictive value of the *N*-glycan score for CRPC was significant, with an area under the curve (AUC) of 0.88 (95% CI = 0.83–0.93, *P* < 0.001) (**C**). The CSS (from serum *N*-glycan analysis until death) were significantly shorter in CRPC patients with higher CRPC *N*-glycan score (>1.7 points) than in those with lower scores (≤1.7 points) (**D**; *P* = 0.028). The longitudinal evaluation of the CRPC *N*-glycan score showed the CRPC *N*-glycan score was greater before-and-after CRPC (n = 5) than that at the baseline (**E**; *P* = 0.048).

**Figure 4:**
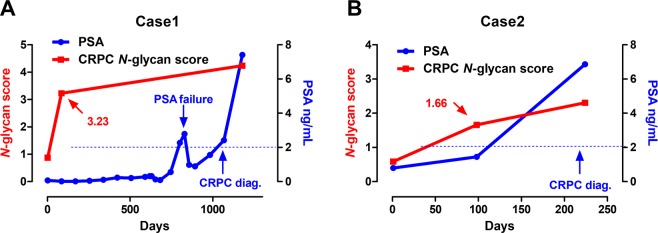
The longitudinal value of the CRPC *N*-glycan score. The case 1 showed early elevation (−985 days) of the CRPC *N*-glycan score before the PSA-based CRPC diagnosis (**A**). Similarly, the case 2 showed early elevation (−126 days) of the CRPC *N*-glycan score before the PSA-based CRPC diagnosis (**B**).

Serum *N*-glycan analysis identified 36 types of benzyloxyamine (BOA)-labeled *N*-glycans (including isomers) that showed quantitative reproducibility in all serum samples (Table S1 and Fig. S1). We observed significant differences in the *N*-glycan profiles between the CRPC and the non-CRPC groups. Of the 36 serum *N*-glycans, we selected nine *N*-glycans for CRPC detection those were significantly greater in the CRPC groups than in the non-CRPC groups **(**Fig. 1 and Table S2). The serum levels of *N*-glycans were significantly associated with the CRPC status including *m/z* 1871 (Fig. 1A), *m/z* 2337 (Fig. 1B), *m/z* 2744 (Fig. 1C), *m/z* 2890 (Fig. 1D), *m/z* 3049 (Fig. 1E), *m/z* 3109 (Fig. 1F), *m/z* 3195 (Fig. 1G), *m/z* 3414 (Fig. 1H), and *m/z* 3719 (Fig. 1I). The representative mass spectra of BPH (Fig. 2A), newly diagnosed PC (Fig. 2B) PC-ADT (Fig. 2C), and CRPC (Fig. 2D) were shown. Based on these results, we obtained the CRPC *N*-glycan score by discriminant analysis to distinguish between CRPC and non-CRPC patients. The formula was obtained as follows: The CRPC *N*-glycan score = (*m/z* 1871 × 0.738) + (*m/z* 2337 × −1.710) + (*m/z* 2744 × 1.304) + (*m/z* 2890 × −0.923) + (*m/z* 3049 × −0.611) + (*m/z* 3109 × −0.622) + (*m/z* 3195 × 1.351) + (*m/z* 3414 × 3.121) + (*m/z* 3719 × −0.011) − 2.709. The Waterfall plot of the CRPC *N*-glycan score showed the hit-rate of the CRPC *N*-glycan score at 85% (Fig. 3A). The *N*-glycan score in the CRPC group was significantly greater than that in the BPH, newly diagnosed PC, and PC-ADT (*P* < 0.001) groups (Fig. 3B). The predictive value of the CRPC *N*-glycan score was significant, with an AUC of 0.88 (95% CI, 0.83–0.93; *P* < 0.001) (Fig. 3C). The Overall survival (OS) from serum *N*-glycan analysis until death was significantly shorter in CRPC patients with greater CRPC *N*-glycan score (>1.7 points) than in those with a lower score (≤1.7 points) (Fig. 3D; *P* = 0.028). We identified five patients who developed CRPC under the longitudinal serum *N*-glycan follow-up. The value of the CRPC *N*-glycan score was greater before-and-after CRPC (n = 5) than that at the baseline (Fig. 3E; *P* = 0.048). The longitudinal evaluation of the CRPC *N*-glycan score showed the early elevation of the *N*-glycan score before the CRPC diagnosis in case 1 (Fig. 4A) and 2 (Fig. 4B).

### *N*-Glycan analysis of Igs andα-1-acid glycoprotein (AGP) as a potential carrier protein

**Figure 5:**
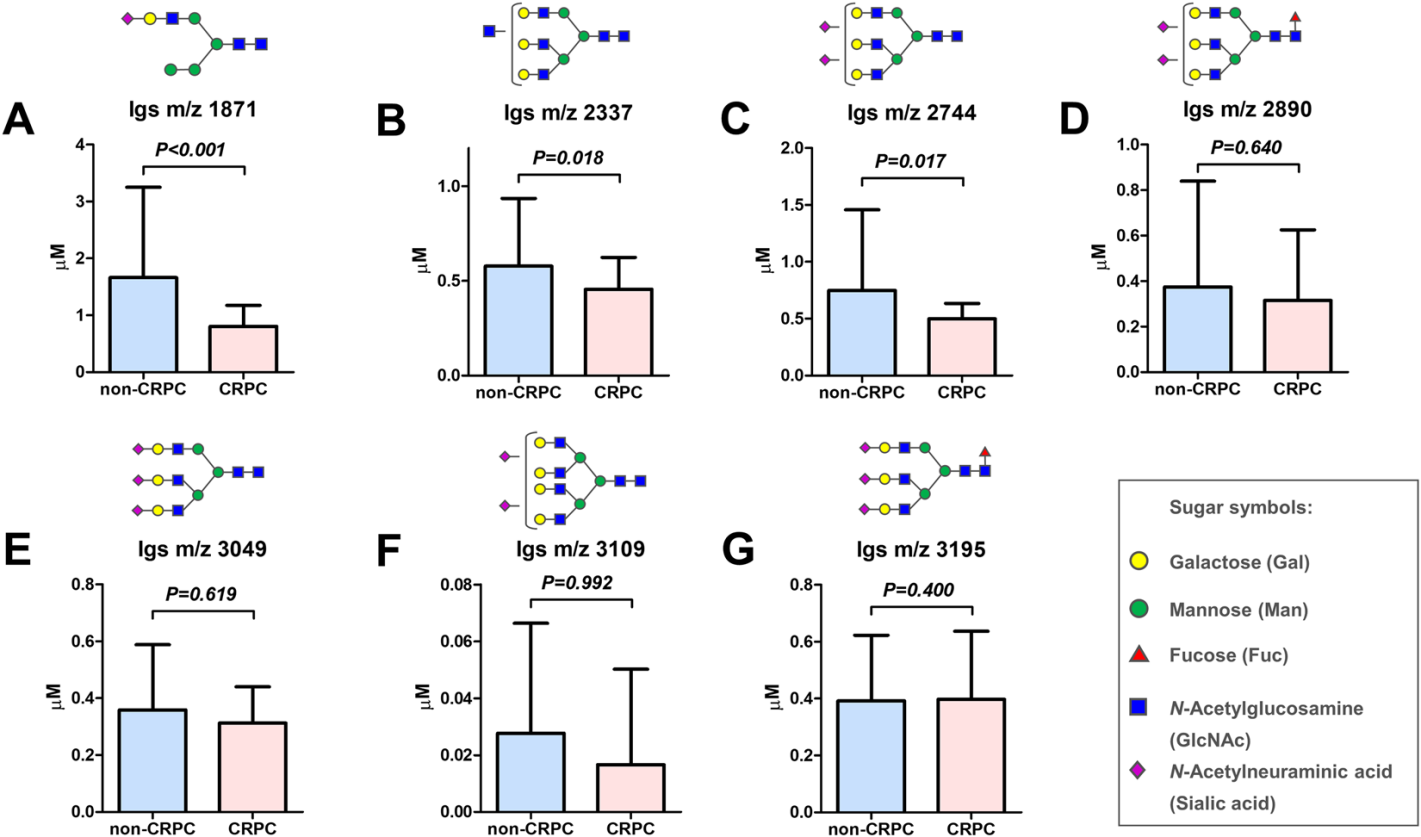
*N*-Glycan analysis of immunoglobulins (Igs). *N*-Glycan profiling in Igs was not consistent with that of whole serum *N*-glycan profiling. The Igs-related *N*-glycans of *m/z* 1871 (**A**), 2337 (**B)**, and 2744 **(C**) were significantly decreased in the CRPC patients than that of non-CPRC patients. There was no significant difference in the *m/z* 2890 (**D**), 3049 (**E**), 3109 (**F**), and 3195 (**G**) between the CRPC and non-CPRC patients. The *N*-glycans of *m/z* 3414, and *m/z* 3719 were not detected on Igs.

**Figure 6:**
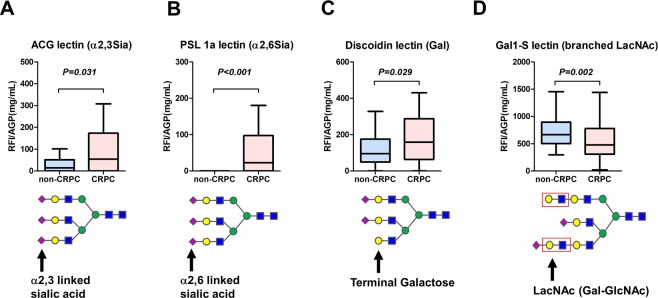
Detection of lectin-reactive glycans on α-1-acid glycoprotein (AGP) using recombinant lectin array chip. The AGP concentration adjusted lectin array analysis showed that terminal α2.3 sialylated glycan (**A**), α2.6 sialylated glycan (**B**), and terminal galactose (**C**), were significantly increased in the CRPC patients. In contrast, branched-LacNAc structure was significantly decreased in the CRPC patients (**D**). RFI: Relative fluorescent intensity.

*N*-Glycan profiling in Igs showed a different pattern than that of whole serum *N*-glycan profiling (Table S3). The Igs-related *N*-glycans of *m/z* 1871, 2337, and 2744 were significantly decreased in the CRPC patients than that of non-CPRC patients (Fig. 5A–C, respectively). There was no significant difference in the *m/z* 2890, 3049, 3109, and 3195 between the CRPC and non-CPRC patients (Fig. 5D–G, respectively). The *N*-glycans of *m/z* 3414, and *m/z* 3719 were not detected on Igs. Thereafter, we investigated the other possible serum carrier protein; AGP, which is an acute-phase serum glycoprotein that possesses five *N*-linked complex type heteroglycan side chains. We detected lectin-reactive *N*-glycans on AGP using recombinant lectin array chip. There was no significant difference in the AGP concentration between the non-CRPC and CRPC groups (1.66 vs 1.52 mg/mL, respectively; *P* = 0.146). The AGP concentration adjusted lectin array analysis showed that terminal α2.3 sialylated glycan (detected by agrocybe cylindracea: ACG lectin) (Fig. 6A), α2.6 sialylated glycan (detected by polyporus squamosus 1a: PSL lectin) (Fig. 6B), and terminal galactose (detected by discoidin lectin) (Fig. 6C), were significantly increased in the CRPC patients. In contrast, branched-LacNAc structure (detected by human galectin1-S: Gal1-S lectin) was significantly decreased in the CRPC patients (Fig. 6D).

**Figure 7:**
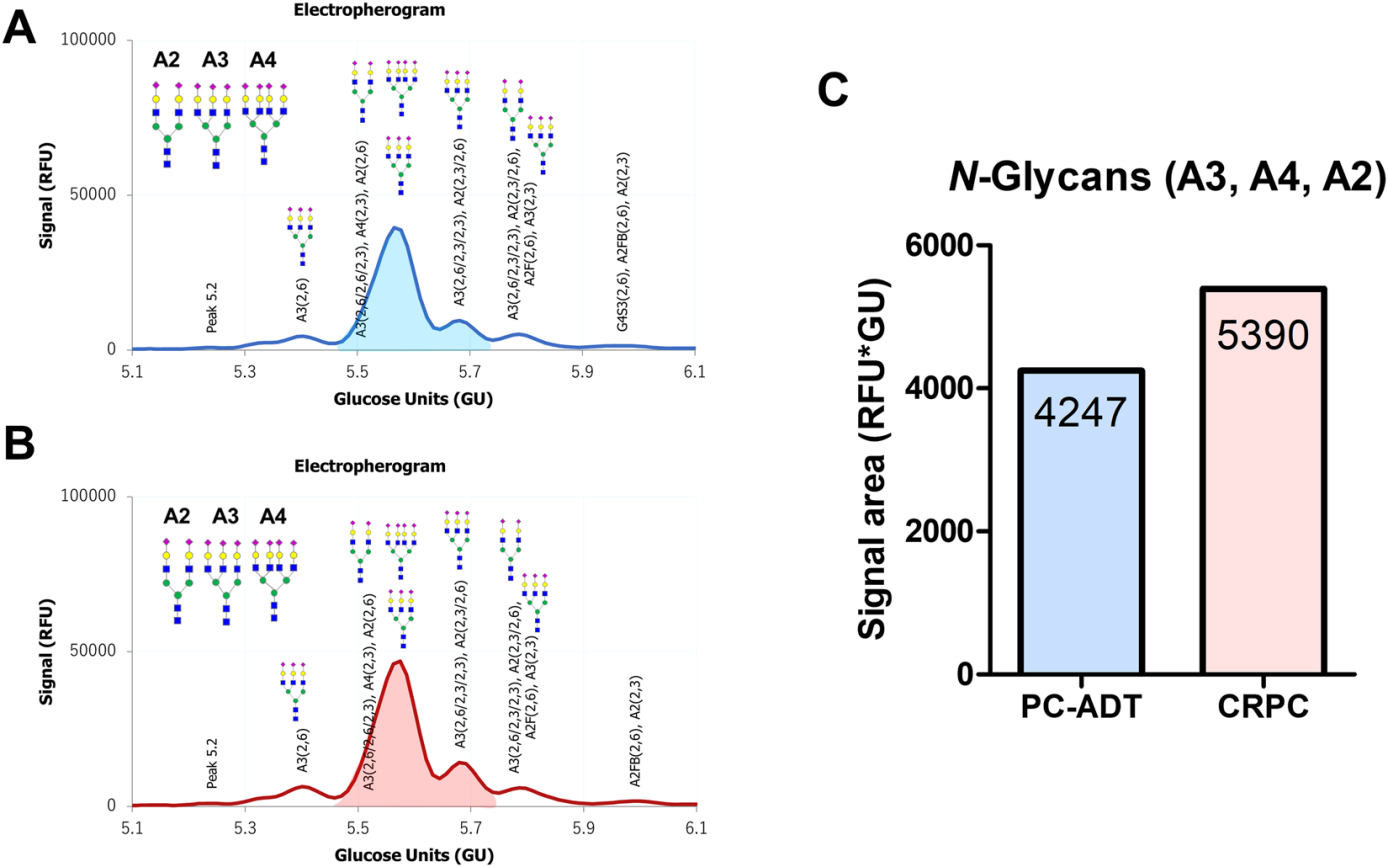
The difference in the serum *N*-glycans on α-1-acid glycoprotein (AGP) between the PC-ADT and CRPC patients using Gly-Q system. Serum *N*-glycans on AGP (pooled serum) were measured between the PC-ADT (**A**) and CRPC (**B**). The signal area of A3, A4, and A2 were higher in the patients with CRPC (5390 RFU * GU) than that of PC-ADT (4247 RFU * GU) (**C**). RFU: Relative Fluorescence Unit, GU: Glucose Unit.

Serum *N*-glycans on AGP (pooled serum) were measured in the PC-ADT (Fig. 7A) and CRPC (Fig. 7B). The signal area of A3, A4, and A2 (amount of *N*-glycans; A3 > A4 > A2) were higher in the patients with CRPC (5390 RFU * GU) than that of PC-ADT (4247 RFU * GU) (Fig. 7C).

## Discussion

We evaluated the diagnostic and prognostic potentials of serum *N*-glycan profiling for CRPC using high-throughput, comprehensive, and quantitative *N*-glycomics that have a potential to distinguish CRPC. Our results demonstrated that the serum levels of *N*-glycans were significantly different between CRPC and non-CRPC patients. The CRPC *N*-glycan score was significantly associated with poor prognosis in CRPC patients. In addition, the carrier protein analysis showed not Igs but AGP was the candidate for tri- and tetra-antennary *N*-glycans. Expression of *N*-glycan branching enzyme genes in CRPC cell lines supports the association between branched *N*-glycan and CRPC.

Although several studies have reported that differences in glycan profiling between diseased and benign states may facilitate the diagnosis or prognosis of diseases, a few studies have investigated the differences in *N*-glycan profiling between healthy male subjects and PC patients^14,21,30^. Kyselova *et al*.^30^ investigated the *N*-glycan profiles (50 types of *N*-glycan) derived from the sera of 10 healthy men in comparison with those from 24 metastatic PC patients. Although the sample size was extremely small, the authors reported that the tri- and tetra-antennary *N*-glycans were significantly greater in the metastatic PC patients than in the healthy subjects. This observation was consistent with our results. In our previous study, we performed a comprehensive *N*-glycan structural analysis and found that tri- and tetra-antennary *N*-glycans (*m/z* 3049 and 3414) were significantly enriched in CRPC patients than in non-CRPC patients and that the expression of *N*-glycan branching enzyme genes (*MGATs*) was significantly upregulated in the CRPC cell lines^14^. In addition, cancer-associated aberrant glycosylation increases the transcription of *MGAT5*, which initiates β1,6GlcNAc branching in tri- and tetra-branched *N*-glycans in PC, that was reported to play an important role in the metastasis^31^. However, not enough evidences are available for the direct association of *N*-glycan branching enzyme genes between prostate cancer cell lines and serum *N*-glycans. Therefore, the relationship of these enzymes between the CRPC cells and serum *N*-glycans needs to be investigated. Further studies are necessary on this issue.

The difference between the present results from our previous results^14^ is notable. Our previous study identified two specific tri- and tetra-antennary *N*-glycans (*m/z* 3049 and 3414) that were significantly enriched in CRPC patients. In the present study, we included nine *N*-glycans and developed the CRPC *N*-glycan score to distinguish between CRPC and non-CRPC patients and evaluated the prognostic value of the CRPC *N*-glycan score. The CRPC *N*-glycan score of >1.7 point was found to be a significant predictor of poor prognosis in patients with CRPC. We found the CRPC *N*-glycan score was significantly different before and after CRPC diagnosis (Fig. 3B). In addition, longitudinal evaluation for the CRPC *N*-glycan score suggested that CRPC *N*-glycan score can detect non-PSA-related changes in patients who potentially developed to CRPC (Fig. 3E) and suggest that the overexpression of specific *N*-glycans may be associated with CRPC. However, the clinical implication of longitudinal evaluation for the *N*-glycan score on CRPC diagnosis may be limited by the small sample size, necessitating further studies.

In this study, we tried to investigate the potential carrier protein of serum *N-*glycans. As previous study suggested that glycan structures of Igs, haptoglobin, and AGP have great potential to serve as cancer diagnosis, prognosis, and treatment monitoring biomarkers to facilitate personalized medicine^21,32^, we evaluated glycan structures of Igs and AGP in serum. First, we hypothesized that serum *N*-glycan profiles might reflect changes in *N*-glycosylation of Igs in CRPC patients because Igs are one of the major *N*-glycosylated proteins in serum. However, the results revealed that the major carrier proteins of CRPC-related aberrant *N*-glycans did not originate from Igs (Fig. 5). Next, we investigated the other possible serum carrier protein. AGP is an acute-phase serum glycoprotein that possesses five *N*-linked complex type heteroglycan side chains, which may be present as biantennary, triantennary, or tetra-antennary structures^21^. We investigated the *N*-glycan profiling on AGP using lectin array analysis. Our results showed that terminal α2.3 sialylated glycan, α2.6 sialylated glycan, and terminal galactose were significantly increased in the CRPC patients (Fig. 6). In addition, capillary electrophoresis-based Gly-Q^TM^ *N*-glycan analysis revealed that tri- and tetra-branched *N*-glycans on AGP were increased in the CRPC patients (Fig. 7). Although we need to improve several technical issues to demonstrate the direct association between the specific *N*-glycans on the AGP and CPRC, these results suggested that terminal sialylated and terminal galactose on AGP were increased in the CRPC patients.

There were several limitations in the present study with regard to the retrospective nature of the study and the small sample size. To validate the proposed predictive biomarkers for CRPC, a prospective study with larger sample size is required. In addition, the predictive potential of serum *N*-glycan as a surrogate biomarker remains unclear due to the un-unified measurement points of serum *N*-glycans. Furthermore, we could not clearly determine the carrier proteins responsible for transporting tri- and tetra-antennary *N*-glycans into the circulation. Also, a lectin array analysis does not confirm the entire structure of *N*-glycans. Despite these limitations, the overexpression of tri- and tetra-antennary *N*-glycans was clearly demonstrated to be a potential biomarker for the detection of CRPC in this study. Future large-scale prospective validation studies may determine the clinical significance of these carbohydrate biomarkers.

In conclusion, the incorporation of glycan biomarkers is a promising approach for improving CRPC detection. Further large-scale prospective studies can help evaluate the utility of these new approaches as well as improve the risk-assessment strategies and clinical outcomes in CRPC patients.

## Methods

### Subject selection

A total of 693 patients with BPH, newly diagnosed PC, PC-ADT, or CRPC were treated at our hospital between June 2007 and December 2018. The serum samples from BPH (n = 287), newly diagnosed PC (n = 289), PC-ADT (n = 57) and CRPC (n = 60) patients were obtained at the time of diagnosis or initial treatment. CRPC was defined by PSA or radiographic progression despite the castrate testosterone level of <50 ng/dL. All samples were stored at −80 °C until use. This study was performed in accordance with the ethical standards of the Declaration of Helsinki and approved by the Institutional Ethics Committee of Hirosaki University Graduate School of Medicine (Authorization Number: 2015-144). Informed consent was obtained from all patients.

### Glycoblotting methods and mass spectrometry

Serum *N*-glycan analysis was performed as described previously by using glycoblotting methods (SweetBlot™; System Instruments, Hachioji, Japan). Briefly, 10 μL of whole serum was subjected to glycoblotting using controlled automated Sweetblot^TM^ (System Instruments). The resulting benzyloxiamine (BOA)-labeled glycans were detected by matrix assisted laser desorption ionization-time of flight mass spectrometry (MALDI-TOF MS) (Ultraflex 3 TOF/TOF Mass Spectrometer; Bruker Daltonics, Bremen, Germany). The composition and structure of the glycans were predicted by using the GlycoMod Tool. The quantitative reproducibility test for SweetBlot was performed as described previously^14^.

### Discriminant analysis for CRPC

To select specific *N*-glycans for the CRPC status, the *N*-glycan levels were analyzed by logistic regression analysis, receiver operating characteristic (ROC) curve, and the area under the curve (AUC). To distinguish CRPC from non-CRPC (BPH, newly diagnosed PC, and PC-ADT) patients, we developed an integrated *N*-glycan biomarker for CRPC using discriminant analysis and the least squares method for a polynomial curve fit. The formula for “the CRPC *N*-glycan score” was developed using a discriminant coefficient. These coefficients can be used to calculate the discriminant score for a given case. The score is calculated in the same manner as a predicted value from a linear regression using standardized coefficients and variables.

### Quantification and purification of the Igs fraction from serum

Ig*s* (IgG1, IgG2, IgG3, IgG4, total IgG, IgM, and IgA) levels of serum were measured by using Bio-plex pro human isotyping 6-plex kit (Bio-rad laboratories, CA, USA) according to instructions. Serum Ig*s* purification was performed using a Zeba^TM^ Spin Desalting Plate and Melon^TM^ Gel Spin Purification Kit (Thermo Fisher Scientific, Waltham, MA, USA) according to instructions. Briefly, serum samples (100 μL) were applied to the center of the Zeba desalting resin equilibrated with PBS and centrifuged at 1000 × *g* for 2 min. The flow-through was collected as buffer-exchanged serum (100 μL) and then applied to the center of the Melon Gel resin equilibrated with purification buffer. After 5 min incubation of Melon Gel resin and buffer-exchanged serum, then the sample was centrifuged at 1000 × *g* for 2 min. Flow-through was collected as a purified Ig*s* fraction. A 10-μL aliquot of the Ig*s* fraction was subjected to our glycoblotting method and mass spectrometry.

### Detection of lectin-reactive glycan on α-1-acid glycoprotein (AGP) using recombinant lectin array chip

To evaluate the *N*-glycans on AGP, we performed a lectin-array analysis. Briefly, 20 μL of serum was diluted by 80 μL of probing buffer. Diluted serum was added to the well of recombinant lectin array chip (Rexxam Co. Ltd., Osaka, Japan). After 70 mins incubation and washing twice, 100 μL of probing buffer containing 1 μg/mL biotinylated anti-AGP antibody (EPR5605) (Abcam, ab134042) was added to well. After 60 mins incubation and washing twice, 100 μL of probing buffer containing 1 μg/mL Cy3 labeled streptavidin (GE-Healthcare, Buckinghamshire, UK) was added to the well. After 30 mins incubation and washing twice, the chip was scanned by utilizing Bio-REX Scan 200 evanescent fluorescence scanner (Rexxam Co., Ltd.). Mean fluorescence intensity of lectin reactive glycan carrying Ig*s* was normalized by total Ig*s* level.

### Detection of *N*-glycan on AGP using Gly-Q Glycan analysis system

To confirm the result of *N*-glycomics of AGP, we compared the *N*-glycan of AGP between the PC-ADT and CRPC patients using a capillary electrophoresis-LED-induced fluorescence based Gly-Q^TM^ *N*-glycan analysis (Prozyme, Inc., CA, USA) combined with Gly-X rapid *N*-glycan preparation method^33^ under controlled automated Sweetblot^TM^ (System Instruments, Tokyo, Japan) machinery, Briefly, 1 mg/mL of AGP from the pooled serum (from 10 patients) and 2 μL of Gly-X denaturant was mixed. Then, 2 μL of *N*-glycanase working solution was added to the denatured samples. After deglycosylation, 5 μL of InstantPC dye solution was added to the deglycosylated samples. The InstantPC Dye and deglycosyalted sample mixture was then loaded onto prewetted Gly-X cleanup plate and applied vaccum to <5 inHg. Then, 100 μL of Gly-X InstantPC eluent added to each well and collected InstanPC-labeled glycan samples into the Collection Plate using vacuum. Finally, InstantQ is a charged *N*-glycan dye that facilitates separation of labeled *N*-glycans on the Gly-Q CE system. Composition and structures of the glycans were analyzed using the Gly-Q Manager software performing automated peak analysis (Relative Fluorescence Unit: RFU and Glucose Unit: GU) and glycan assignments from the glycan library.

### Statistical analysis

Statistical analysis was performed using SPSS ver. 24.0 (SPSS, Inc., Chicago, IL, USA) and GraphPad Prism 5.03 (GraphPad Software, San Diego, CA, USA). The intergroup differences were tested by Student’s *t*-test or the Mann–Whitney U-test. The differences among three or more groups were analyzed using the Kruskal–Wallis test. The optimal cutoff points for CRPC detection and poor prognosis were calculated using the following formula: (1−sensitivity)^2^ + (1−specificity)^2^. *P* < 0.05 was considered significant. The overall survival (OS) from serum *N*-glycan analysis until death was estimated using the Kaplan–Meier curve and the log–rank test.

### Informed consent

All participants provided written informed consent.

## Supplementary information

Supplementary information

## Data Availability

The minimal data set of the present study is available on request.
