# Supplementary material for: Serum N-glycan profiling is a potential biomarker for castration-resistant prostate cancer
Source: Sci Rep. 2019 Nov 14;9:16761. doi: 10.1038/s41598-019-53384-y (PMC6856113; doi:10.1038/s41598-019-53384-y)
Supplement: Supplementary file 1 — Supplementary information [file 41598_2019_53384_MOESM1_ESM.pdf]

Serum *N*-glycan profiling is a potential biomarker for castration-resistant prostate cancer

Teppei Matsumoto[1], Shingo Hatakeyama*[1], Tohru Yoneyama[2], Yuki Tobisawa[1], Yusuke Ishibashi[1], Hayato Yamamoto[1], Takahiro Yoneyama[2], Yasuhiro Hashimoto[1], Hiroyuki Ito[3], Shin-Ichiro Nishimura[4], Chikara Ohyama[1,2]

[1]Department of Urology, Hirosaki University Graduate School of Medicine, Hirosaki 036-8562, Japan

[2]Department of Advanced Transplant and Regenerative Medicine, Urology, Hirosaki University Graduate School of Medicine, Hirosaki 036-8562, Japan

[3]Department of Urology, Aomori Rosai Hospital, Hachinohe 031-8551, Japan

[4] Graduate School of Life Science, Frontier Research Centre for Advanced Material and Life Science, Hokkaido University, Sapporo 060-0808, Japan

* Corresponding author: Shingo Hatakeyama, MD

Department of Urology, Hirosaki University Graduate School of Medicine, 5 Zaifu-chou, Hirosaki 036-8562, Japan, Tel: 81-172-39-5091, Fax: 81-172-39-5092, E-mail: shingoh@hirosaki-u.ac.jp

Supplementary figure legend

Fig. S1: A representative structure of serum *N*-glycans

Serum *N*-glycan analysis identified 36 types of benzyloxyamine (BOA)-labeled *N*-glycans that showed quantitative reproducibility in all serum samples.

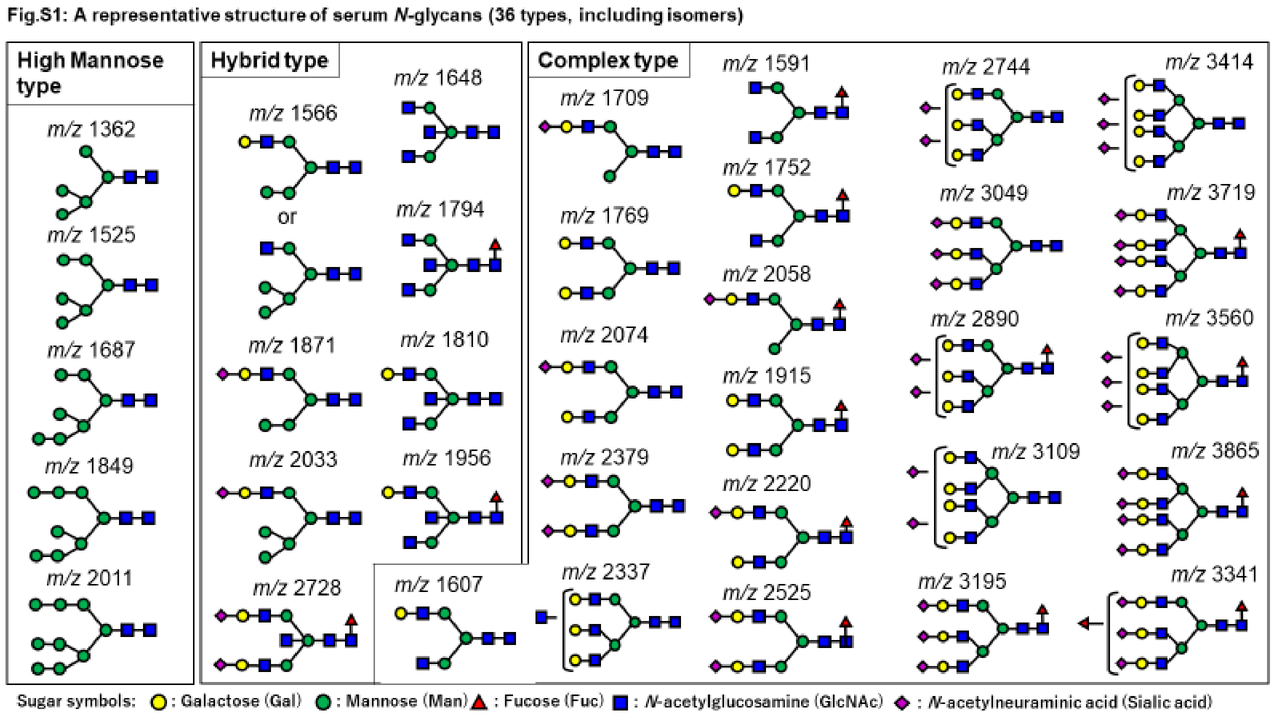

Supplementary tables

Table S1: Thirty-Six types of *N*-glycans demonstrated quantitative reproducibility in all samples.

The *m/z* 2348.9 is the internal standard, disialo-galactosylated biantennary *N*-glycan, that contains amidated sialic acids (A2 amide glycans). Compositional annotations and putative structures are shown as abbreviations.

Peak No.	*m/z*	Composition
1	1362.5	(Hex)2 + (Man)3(GlcNAc)2
2	1524.5	(Hex)3 + (Man)3(GlcNAc)2
3	1565.5	(Hex)5 + (HexNAc)3
4	1590.6	(HexNAc)2(dHex)1 + (Man)3(GlcNAc)2
5	1606.6	(Hex)1(HexNAc)2 + (Man)3(GlcNAc)2
6	1647.6	(HexNAc)3 + (Man)3(GlcNAc)2
7	1686.6	(Hex)4 + (Man)3(GlcNAc)2
8	1708.6	(Hex)1(HexNAc)1(NeuAc)1 + (Man)3(GlcNAc)2
9	1752.6	(Hex)1(HexNAc)2(dHex)1 + (Man)3(GlcNAc)2
10	1768.6	(Hex)2(HexNAc)2 + (Man)3(GlcNAc)2
11	1793.7	(HexNAc)3(dHex)1 + (Man)3(GlcNAc)2
12	1809.7	(Hex)1(HexNAc)3 + (Man)3(GlcNAc)2
13	1848.6	(Hex)5 + (Man)3(GlcNAc)2
14	1870.7	(Hex)2(HexNAc)1(NeuAc)1 + (Man)3(GlcNAc)2
15	1914.7	(Hex)2(HexNAc)2(dHex)1 + (Man)3(GlcNAc)2
16	1955.7	(Hex)1(HexNAc)3(dHex)1 + (Man)3(GlcNAc)2
17	2010.7	(Hex)6 + (Man)3(GlcNAc)2
18	2032.7	(Hex)3(HexNac)1(NeuAc)1 + (Man)3(GlcNAc)2
19	2057.8	(Hex)1(HexNAc)2(dHex)1(NeuAc)1+ (Man)3(GlcNAc)2
20	2073.8	(Hex)2(HexNAc)2(NeuAc)1+ (Man)3(GlcNAc)2
21	2219.8	(Hex)2(HexNAc)2(dHex)1(NeuAc)1 + (Man)3(GlcNAc)2
22	2336.9	(Hex)3(HexNAc)4 + (Man)3(GlcNAc)2
**23**	**2348.9**	**Internal standard (BOA-labeled A2 amide)**
24	2378.9	(Hex)2(HexNAc)2(NeuAc)2 + (Man)3(GlcNAc)2
25	2524.9	(Hex)2(HexNAc)2(dHex)1(NeuAc)2 + (Man)3(GlcNAc)2
26	2727.9	(Hex)2(HexNAc)3(dHex)1(NeuAc)2 + (Man)3(GlcNAc)2
27	2743.9	(Hex)3(HexNAc)3(NeuAc)2 + (Man)3(GlcNAc)2
28	2890.1	(Hex)3(HexNAc)3(dHex)1(NeuAc)2 + (Man)3(GlcNAc)2
29	3049.1	(Hex)3(HexNAc)3(NeuAc)3 + (Man)3(GlcNAc)2
30	3109.1	(Hex)4(HexNAc)4(NeuAc)2 + (Man)3(GlcNAc)2
31	3195.2	(Hex)3(HexNAc)3(dHex)1(NeuAc)3 + (Man)3(GlcNAc)2

32	3341.2	(Hex)3 (HexNAc)3 (Deoxyhexose)2 (NeuAc)3 + (Man)3(GlcNAc)2
33	3414.2	(Hex)4(HexNAc)4(NeuAc)3 + (Man)3(GlcNAc)2
34	3560.3	(Hex)4(HexNAc)4(dHex)1(NeuAc)3 + (Man)3(GlcNAc)2
35	3719.3	(Hex)4(HexNAc)4(NeuAc)4 + (Man)3(GlcNAc)2
36	3865.4	(Hex)4(HexNAc)4(dHex)1(NeuAc)4 + (Man)3(GlcNAc)2

Hex: hexose; HexNAc: *N*-acetylhexosamine; dHex: deoxyhexose.

Table S2 Comparison of serum *N*-glycans between non-CRPC and CRPC (†, *P* < 0.001)

m/z	non-CRPC	CRPC	*P value*
**1362**	1.624	1.570	*0.539*
**1525**	1.445	1.513	*0.356*
**1566**	2.072	1.929	*0.297*
**1591**	2.618	2.393	*0.774*
**1607**	2.520	2.388	*0.764*
**1648**	2.071	2.225	*0.161*
**1687**	1.349	1.464	*0.002*
**1709**	1.434	1.563	*0.009*
**1753**	2.104	1.966	*0.191*
**1769**	2.317	2.262	*0.318*
**1794**	2.856	3.090	*0.280*
**1810**	2.009	2.206	*0.341*
**1849**	1.463	1.525	*0.136*
**1871**†	1.666	2.041	*<0.001*
**1915**	2.113	1.969	*0.853*
**1956**	2.321	2.448	*0.398*
**2011**	1.512	1.549	*0.011*
**2033**	1.615	1.781	*0.012*
**2058**	2.282	2.271	*0.975*
**2074**	1.399	1.494	*0.005*
**2220**	1.902	1.877	*0.809*
**2337**†	1.341	1.449	*<0.001*
**2379**	1.361	1.459	*0.007*
**2525**	1.626	1.708	*0.009*
**2728**	1.871	1.846	*0.743*
**2744**†	1.374	1.967	*<0.001*
**2890**†	1.419	1.891	*<0.001*
**3049**†	1.382	1.879	*<0.001*
**3109**†	1.257	1.874	*<0.001*
**3195**†	1.374	1.782	*<0.001*
**3341**	0.000	0.741	*0.033*
**3414**†	1.218	1.803	*<0.001*
**3560**	1.156	1.425	*0.013*
**3719**†	1.168	1.617	*<0.001*
**3865**	1.010	1.212	*0.011*

Table S3 Comparison of serum *N*-glycans on immunoglobulins (Igs) between non-CRPC and CRPC (†, *P* < 0.001, N/A: not applicable)

m/z (Igs)	non-CRPC	CRPC	*P value*
**1362**	0.688	0.418	*0.008*
**1525**	0.363	0.304	*0.024*
**1566**	0.000	0.000	*N/A*
**1591**	4.531	2.807	*0.012*
**1607**	3.166	1.821	*0.002*
**1648**	2.188	1.512	*0.015*
**1687**	0.000	0.000	*0.050*
**1709†**	0.556	0.414	*<0.001*
**1753**	2.804	1.919	*0.009*
**1769**	0.000	0.000	*0.036*
**1794**	4.962	2.851	*0.014*
**1810**	1.774	1.245	*0.013*
**1849**	0.681	0.353	*0.004*
**1871†**	1.113	0.740	*<0.001*
**1915**	2.637	1.710	*0.019*
**1956**	2.970	1.785	*0.008*
**2011**	0.966	0.602	*0.006*
**2033†**	0.544	0.000	*<0.001*
**2058†**	2.039	1.699	*<0.001*
**2074†**	0.518	0.358	*<0.001*
**2220†**	1.159	0.959	*<0.001*
**2337**	0.515	0.454	*0.010*
**2379†**	0.266	0.203	*<0.001*
**2525†**	0.572	0.478	*<0.001*
**2728**	0.918	0.774	*0.003*
**2744**	0.557	0.489	*0.002*
**2890**	0.000	0.440	*0.397*
**3049**	0.305	0.286	*0.152*
**3109**	0.000	0.000	*0.667*
**3195**	0.407	0.350	*0.902*
**3341-3865**	0.000	0.000	*N/A*
